# Blood Metabolic Profile and Hemogram Across Lactation in an Extensively Managed Flock of Dalmatian Pramenka Ewes: Influence of Stage of Lactation and Litter Size

**DOI:** 10.3390/metabo16090668

**Published:** 2026-09-10

**Authors:** Zvonko Antunović, Lucija Bronić, Željka Klir Šalavardić, Mislav Đidara, Luka Šramek, Josip Novoselec

**Affiliations:** Department for Animal Production and Biotechnology, Faculty of Agrobiotechnical Sciences Osijek, University of J. J. Strossmayer, V. Preloga 1, 31000 Osijek, Croatia; zantunovic@fazos.hr (Z.A.); mdidara@fazos.hr (M.Đ.); lsramek@fazos.hr (L.Š.); jnovoselec@fazos.hr (J.N.)

**Keywords:** Dalmatian Pramenka ewes, blood metabolic profile, hemogram, lactation, litter size

## Abstract

**Background/Objectives**: Determination of blood metabolic profile and hemogram are particularly valuable in animals kept in extensive and semi-intensive farming systems, as within those systems, it is not possible to properly monitor animals. This research investigated changes in the hemogram indicators and blood metabolic profile of Dalmatian Pramenka ewes by comparing different lactation stage and litter size. **Methods**: This study investigated changes in the hemogram indicators and blood metabolic profile of 33 Dalmatian Pramenka ewes during lactation as influenced by the lactation stage (taken on the 40th, 70th and 100th day of lactation) and the litter size (1 or 2 lambs in litter). Referring to the litter size, 11 ewes had twins, and 22 ewes had one lamb in litter. **Results**: This study confirmed the significant influence of stage of lactation on the blood metabolic profile (minerals: Ca, P-inorganic, Mg, Fe, metabolites: urea, glucose, triglycerides, ALB, GLOB and A/G ratio, and ALT enzyme) and most of the hemogram indicators (RBC and WBC content of HGB and HCT and portion of monocytes) in the blood of lactating ewes. The influence of litter size on the hemogram and blood metabolic profile was insignificant, yet its only statistically significant effect was determined for urea concentrations in the blood of ewes. **Conclusions**: When planning quality monitoring procedures of a herd, a blood metabolic profile and hemogram of the animals should be implemented as routine herd-monitoring tools for indigenous sheep breeds raised under extensive production systems.

## 1. Introduction

Metabolic profile refers to analytical blood tests that measure the biochemical and hematological indicators of blood [1]. A hemogram includes a blood erythrogram and leukogram, while biochemical indicators refer to concentrations of metabolites, minerals, and enzyme activity in blood. Tracking changes in the metabolic profile of ruminants’ blood with hemograms provides reliable data for determining the health status, nutritional status, and welfare of animals [2,3]. Determination of blood metabolic profile is particularly valuable in animals kept in extensive and semi-intensive farming systems, as within those systems, it is not possible to properly monitor animals (i.e., sheep nutrition) [4,5]. There are numerous factors that influence the hemogram indicators and metabolic profile of sheep’s blood, such as nutrition, physiological status, age, breeding conditions, climate, applied laboratory diagnostics and selected analytical devices, the convenience of taking samples, etc. In demanding production phases, such as lactation, it is necessary to additionally monitor animals because of the higher risk of health disorders and low-quality nutrition, which are often beyond the influence of farmers [6,7]. Furthermore, blood analysis not only provides information on the reproduction and production performance, but also serves as an indicator of organism adaptability [8]. There have not been many studies on the impact of litter size on the metabolic blood profile and hemogram of ewes during lactation. Litter size (LS), defined as the number of lambs born per ewe lambing, is of zoological and economical importance if considering survival of the species and supply of sheep products [9,10]. The influence of litter size on blood metabolic profile and hemogram has been researched more in pregnant sheep, and it was established that there is a significant influence of litter size in ewes carrying twins, as they had a significantly higher number of RBC and WBC as well as a higher content of HGB, PCV, and MCH, and a higher portion of lymphocytes and granulocytes than pregnant sheep carrying a single fetus [11,12]. Yenlimez et al. [13] did not confirm a significant effect of litter size on the hematological parameters, but they reported on the significant variations in biochemical indicators in the blood of pregnant sheep carrying twins (BHB, ALT, AST). Still, there is not enough research that has examined the effect of litter size on the blood metabolic profile and hemogram in lactating ewes [14]. There are approximately 521,000 sheep raised in the Republic of Croatia [15]. The Dalmatian Pramenka is an indigenous Croatian sheep breed. It represents the largest population of indigenous Pramenka sheep currently raised under extensive production systems in Croatia. It is a late-maturing breed that is highly resilient, agile, and well-adapted to the climatic conditions of the region, including high summer temperatures, drought, strong winds, and in some areas, low winter temperatures. Dalmatian Pramenka sheep are primarily raised on natural pastures within the sub-Mediterranean karst region where the Festuco-Koelerietum splendenti and Stipo-Salvetium officinalis pasture communities are the most significant, and they are particularly adept at grazing on shrubs, being adapted to the local ecological conditions of rocky pastures [16]. In the study by Širić et al. [17], the average body weight of Dalmatian Pramenka sheep was 38.56 kg, classifying the breed among the smaller Mediterranean sheep breeds. Milk production during the lactation period, which lasts for 150–180 days, ranges from 50 to 100 kg [18]. The fleece is of an open type, consisting of pointed locks, and the wool is usually white, however, some animals have black wool, while brown and grey fleece colors occur less frequently. The annual wool yield is 1–2 kg per ewe and 2–3 kg per ram. Fertility is 120–140% [18]. The breed has been produced for meat; as of 2023, Dalmatian Pramenka lamb received the EU label of Protected Designation of Origin—PDO.

Results of the hemograms and blood metabolic profiles of lactating Dalmatian Pramenka ewes are more difficult to compare with other results due to the shorter lactation period of Dalmatian Pramenka ewes. Determining the indicators of the hemogram and blood metabolic profile is desirable in monitoring herd quality. The research hypothesis is that stage of lactation will affect the hemogram and blood metabolic profile of ewes, but the litter size will not have significant effect on most of the investigated blood indicators in lactating Dalmatian Pramenka ewes.

This research investigates changes in the indicators of blood metabolic profile and hemogram across lactation in an extensively managed flock of Dalmatian Pramenka ewes by comparing different stages of lactation and litter size.

## 2. Materials and Methods

### 2.1. Location of Research, Selection, and Measurement of Ewes

This study was performed on Dalmatian Pramenka ewes kept at the Bronić Agricultural Family Farm in Oklaj, Šibenik-Knin County, Croatia. Thirty-three clinically healthy ewes were selected from a herd of 120 sheep. Referring to the litter size, 11 ewes had twins, and 22 ewes had one lamb in litter. Ewes were monitored during their lactation and kept together with their lambs. Ewes were approximately 4 years old, in the 3rd lactation, in good health and physical shape. The ewes grazed on natural Mediterranean pasture during the day and ate oats (500 g/day/ewes) and hay (1.5 kg/day/ewes) upon returning to the barn. Freshwater and salt licks were provided ad libitum. Pasture sampling was carried out on the same day as sheep blood sampling. Samples of the feedstuffs (pasture, hay and oat) were taken immediately after mixing and analyzed at the Central Analytical Agrobiotechnical Unit of the Faculty of Agrobiotechnical Sciences in Osijek. Afterward, the feedstuffs were dehydrated and finely ground using an ultra-centrifugal mill free of heavy metals (Microtron MB 550; Kinematica, Luzern, Switzerland). The feedstuffs’ chemical composition was determined using standard methods of the AOAC [19]. Neutral detergent fiber (NDF), acid detergent fiber (ADF) and lignin were determined using the FibreBag system (C. Gerhardt GmbH & Co. KG, Königswinter, Germany). NDF was determined according to Van Soest et al. [20], whereas ADF and lignin were determined according to AOAC Official Method 973.18 [19]. The chemical composition of the feedstuffs are shown in Table 1. Anthelmintic treatments were carried out regularly on the farm during the second half of pregnancy (10th October) with Abantel^®^ 500 mg/20 mg (Genera d.d., Kalinovica, Croatia), giving each sheep one pill. The average body weight of the ewes at the beginning and end of the study were 47.90 kg and 43.21 kg, respectively, and the average body condition score (BCS) was 3.08 and 2.41, respectively. The body condition score of the Dalmatian Pramenka ewes was carried out according to Russel [21], with scores from 1 to 5.

### 2.2. Blood Sampling Procedure and Determination of Hemogram and Blood Metabolic Profile

Sampling of blood from the same ewes was conducted during different stages of lactation (on the 40th, 70th, and 100th day of lactation (±5 days)). Blood sampling from sheep was performed in the morning at 8 am for all measurements before grazing. Ninety-nine blood samples were collected from 33 ewes at three blood sampling time points, and 31 different blood metabolic and hematological parameters were analyzed, which resulted in a total of 3069 individual measurements. Blood was taken into sterile vacuum tubes Venoject^®^ (Sterile Terumo Europe, Leuven, Belgium), which contained an anticoagulant (i.e., ethylenediaminetetraacetic acid (EDTA)). After sampling, blood was cooled down to +4 °C and transferred to the laboratory at the Faculty of Agrobiotechnical Sciences Osijek, where hemogram indicators were determined the same day and blood smears were made to determine the differential blood count. A hemogram includes the determination of erythrogram and leukogram by determining the blood cells in whole blood (WBC—white blood cells and RBC—red blood cells), the content of hematocrit (HCT), hemoglobin (HGB), the mean corpuscular volume (MCV), the mean corpuscular hemoglobin MCH), and the mean corpuscular hemoglobin concentration in the erythrocytes (MCHC) as well as differential blood counts. A Sysmex PocH-100iV automated three-differential hematology analyzer (Sysmex Europe GmbH, Hamburg, Germany) was used for analysis. The percentage shares of neutrophils, lymphocytes, basophils, eosinophils, and monocytes as the leukocyte subpopulations were determined from the differential blood count according to the Pappenheim stain. Blood samples were taken into serum separation tubes (Vacutube, Vodice, Slovenia) and centrifuged for 10 min at 1609.92× *g* in a ROTOFIX 32A centrifuge (Hettich GmbH & Co. KG, Tuttlingen, Germany) to separate the serum, which was then frozen at −80 °C. After the end of the study (day 3), all frozen samples were thawed, and on the same day, biochemical indicators in the blood serum of the studied sheep were analyzed in one set.

An automatic Olympus AU 400 (Olympus, Tokyo, Japan) device was used to analyses the ewes’ blood serum for biochemical indicators (i.e., concentrations of metabolites: glucose and urea, as well as total protein (TP), albumin (ALB), cholesterol, HDL-cholesterol (high-density lipoprotein), LDL-cholesterol (low-density lipoprotein), triglycerides; content of some minerals (Ca, P-inorganic, Mg and Fe), and the enzyme activity of ALT (alanine aminotransferase), AST (aspartate aminotransferase), GGT (γ-glutamyl transferase, ALP (alkaline phosphatase), and CK (creatine kinase)). The content of globulin (GLOB) was expressed as the difference between the total protein content and total albumin. The A/G ratio was obtained by dividing the albumin and globulin.

### 2.3. Statistical Analysis

The normality of the distribution of variables was assessed by the Shapiro–Wilk test (PROC UNIVARIATE). Descriptive statistics were performed, and the values are shown as arithmetic mean, median, standard deviation, and interquartile range, while 95% and 99% confidence intervals were calculated. The data distribution was additionally evaluated with a Q–Q plot. In the analyzed hematological indicators, most of the variables did not follow a normal distribution, with the exception of MCH, while among the biochemical indicators, some of the variables (GGT, HDL, CK, ALP, AST, TRIG, Fe, urea, AG) also showed a deviation from the normal distribution.

For normally distributed data, GLM repeated measures was applied to test the influence of the lactation stage and litter size of ewes during lactation. Tukey’s test was used to compare the means, and the established differences between the groups were marked as significant at the *p* < 0.05 level.

For the analysis of data that did not follow a normal distribution, a rank-transformation of the data was carried out, after which a GLM repeated measurement was applied on the ranked data. Lactation stage and litter size were included in the model as fixed effects, along with their interaction, while animal was defined as the subject. For post hoc comparisons, pairwise comparisons of least square means (LSMEANS) with Tukey’s correction for multiple comparisons were used. Statistical data were processed in SAS^®^ 9.4 (SAS Institute Inc., Cary, NC, USA).

## 3. Results

### 3.1. Influence of Stage of Lactation and Litter Size on Hemogram of Dalmatian Pramenka Ewes

**Figure 1:**
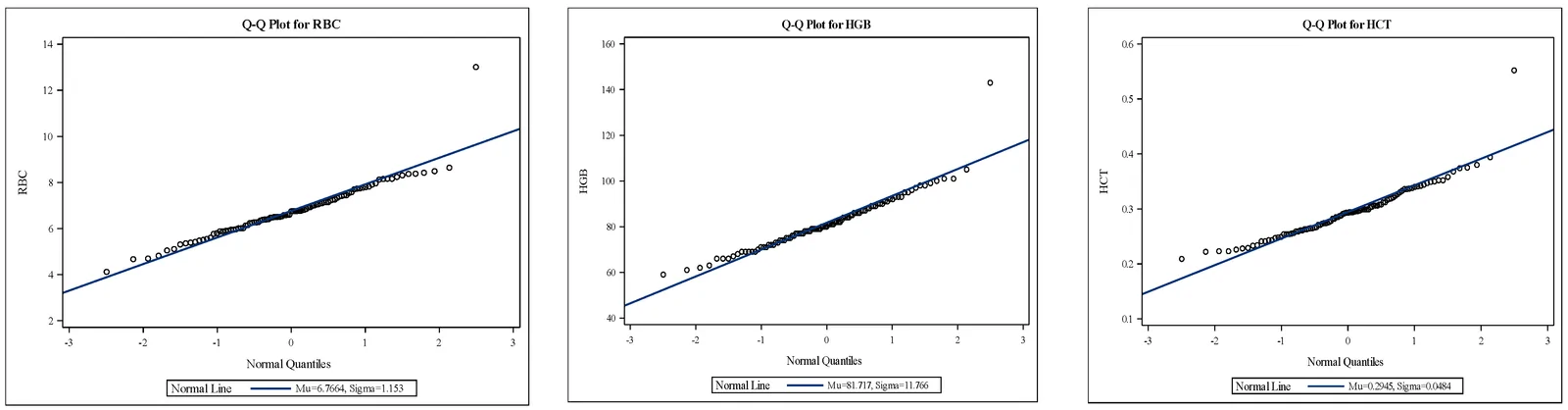

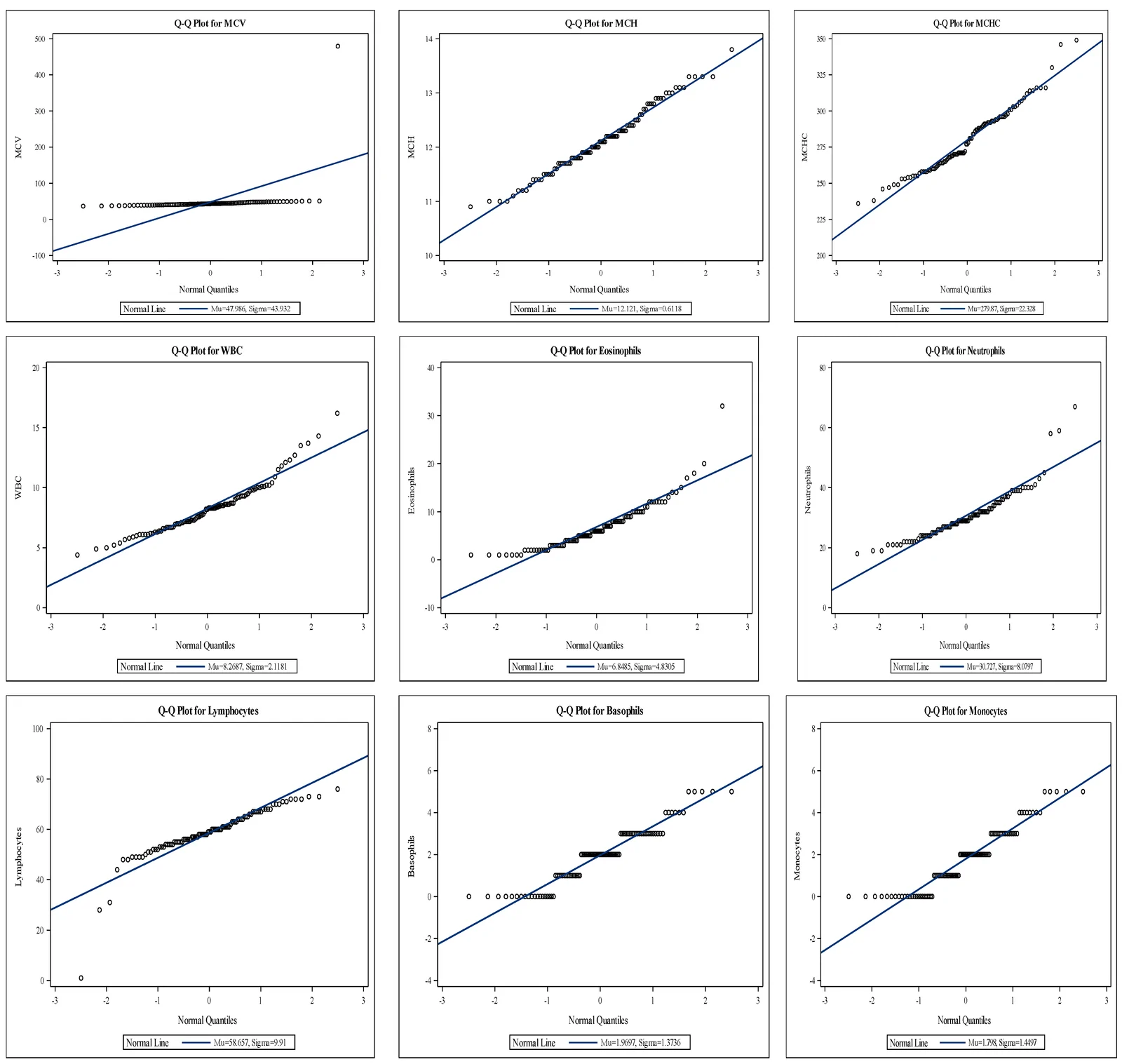
Q–Q plots of the deviation of the obtained values for hemogram of lactating Dalmatian Pramenka ewes. Q–Q plots 1–12 also indicate deviations from the normality of the distribution for the investigated erythrogram and leukogram indicators that make up the hemogram of Dalmatian Pramenka ewes.

In Table 2, the descriptive statistics are presented at the 95% and 99% confidence intervals for lower and upper reference values and distribution for the hemogram indicators of lactating Dalmatian Pramenka ewes. Figure 1 presents the Q–Q plots of the deviation of the obtained values for the hemogram of lactating Dalmatian Pramenka ewes.

Descriptive statistics, including the mean and median values of the hemogram blood indicators of Dalmatian Pramenka ewes, indicated deviation from the reference values for erythrogram and leukogram indicators, and in total, three of them were below (RBC, HGB, MCHC), two indicators were slightly higher (MCV and basophils), and the rest (7) were within the reference values (Table 2). The 95% and 99% confidence intervals of the reference values also followed the stated deviations from the reference values. Only MCH in the blood of Dalmatian Pramenka ewes during lactation showed a normal distribution of hemogram indicators.

Analysis of data contained in Table 3 showed a significant decrease in RBC and the content of HGB and HCT in the blood of Dalmatian Pramenka ewes (*p* < 0.0001) on the 100th day of lactation compared to the 40th and 70th days of lactation. Furthermore, this study found no significant effect of litter size or the interaction between SL × LS on the erythrogram indicators in the blood of Dalmatian Pramenka ewes during lactation (*p* > 0.05).

Analysis of most of the leukogram indicators of Dalmatian Pramenka ewes (Table 4) revealed a significant increase on the 70th lactation day compared to the 40th day, while 100th day their number decreased insignificantly. A significant increase in WBC (*p* = 0.002) on the 70th day and monocytes (*p* = 0.044) on the 100th day of lactation were found compared to the 40th day of lactation. Furthermore, in this study, neither the litter size nor the interaction between SL × LS had an influence on the leukogram indicators in the blood of Dalmatian Pramenka ewes during lactation.

### 3.2. Influence of Lactation Stage and Litter Size on the Blood Metabolic Profile of Dalmatian Pramenka Ewes

**Figure 2:**
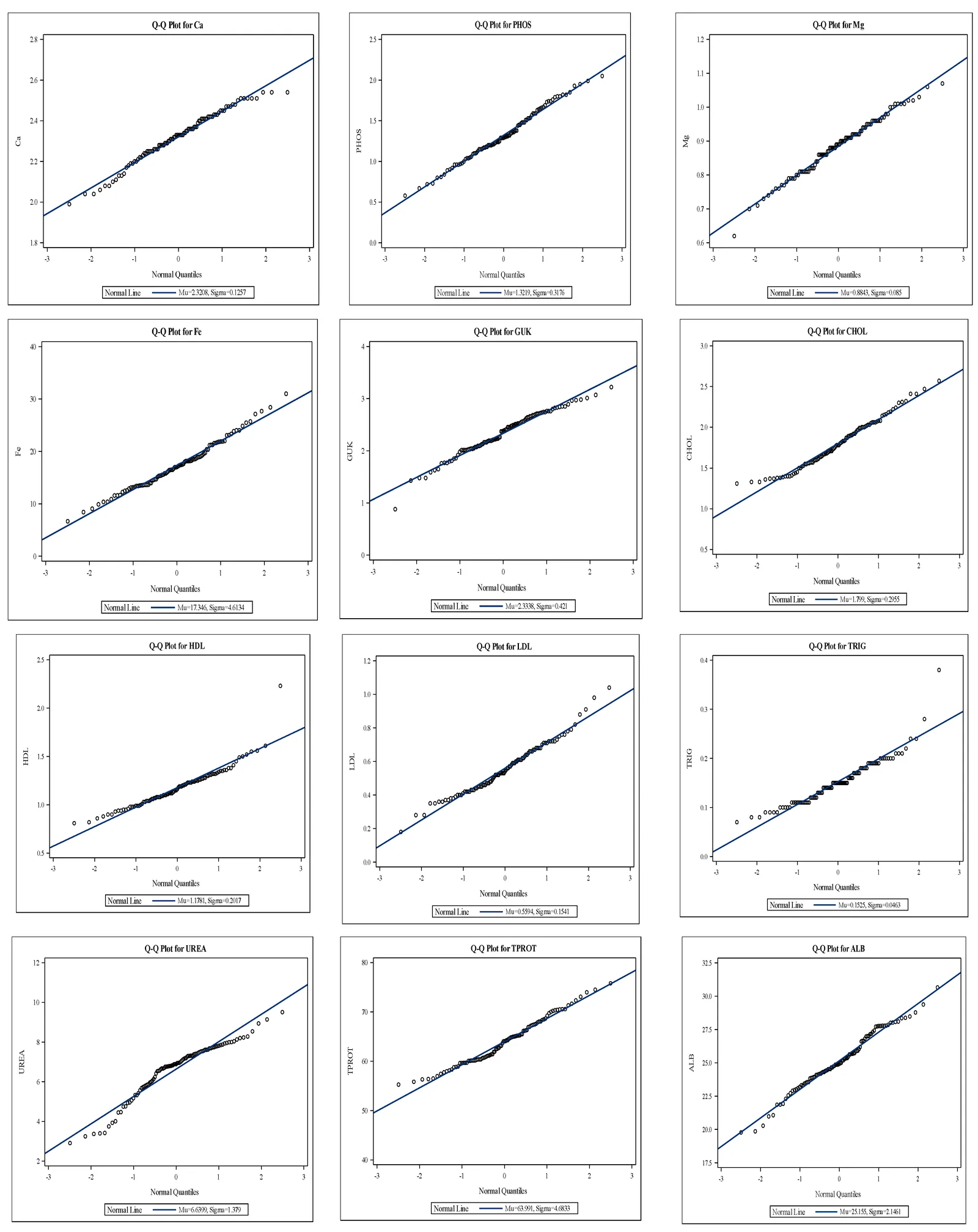

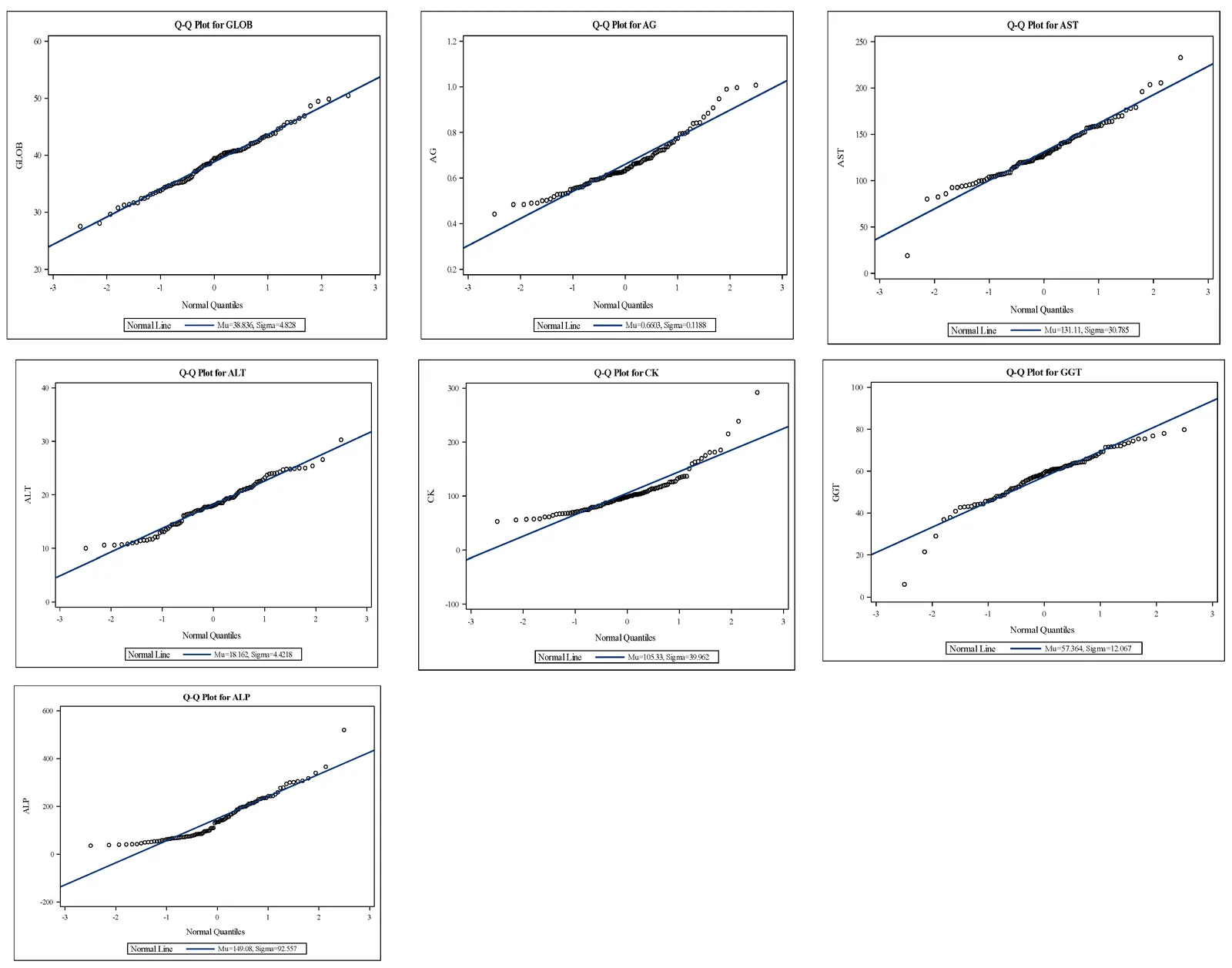
Q–Q plots of the deviation of the obtained values for the blood metabolic profile of lactating Dalmatian Pramenka ewes. Q–Q plots 13–31 also indicate deviations from the normality of the distribution for the investigated indicators of mineral content, metabolites, and enzymes that make up the blood metabolic profile of Dalmatian Pramenka ewes.

In Table 5, descriptive statistics are presented, as well as the 95% and 99% confidence intervals for the lower and upper reference values and distribution for the blood metabolic profile indicators of lactating Dalmatian Pramenka ewes. Figure 2 presents the Q–Q plots of the deviation of the obtained values for the blood metabolic profile of lactating Dalmatian Pramenka ewes.

Descriptive statistics, including the mean and median values of blood indicators of the blood metabolic profile of Dalmatian Pramenka ewes, indicate a smaller deviation from the reference values for the concentrations of all four investigated minerals (Ca, P-inorganic, Mg and Fe) and glucose, higher values of LDL-cholesterol and activity of GGT, and the remaining indicators (12) were within the reference values (Table 5). The largest variations (Sd) were recorded in enzyme activities. Confidence intervals for 95% and 99% of the reference values also followed the stated deviations from the reference values. Most blood metabolic profile indicators of the Dalmatian Pramenka ewes showed a normal distribution, except for Fe, metabolites for urea, HDL-cholesterol, triglycerides, A/G, and enzymes for ALP, CK, ALP, and AST.

The influence of lactation stage and litter size on the blood mineral concentrations of lactating Dalmatian Pramenka ewes is shown in Table 6. A significant decrease was established for concentrations of Ca and Fe as well as an increase in the concentrations of inorganic P and Mg as lactation progressed (*p* < 0.05). Furthermore, this study did not prove a significant influence of litter size, nor of the interaction between SL × LS on mineral concentrations in the blood of Dalmatian Pramenka ewes during lactation (*p* > 0.05).

Analysis of data contained in Table 7 confirmed that the lactation stage had a significant influence on the majority of the determined concentrations of metabolites, except for most indicators of lipid status. As lactation progressed, a significant increase in glucose concentration was found on the 70th day, and then a further decrease on the 100th day of lactation (*p* < 0.0001). There was also the reverse process through an increase in triglyceride concentrations (*p* = 0.0001) in the blood of lactating Dalmatian Pramenka ewes. When compared to the 40th day of lactation, a significant increase in the concentrations of urea and globulin was determined on the 70th and 100th lactation days, but also the reverse process was observed through a significant decrease in ALB and the A/G ratio in the blood of ewes on days 70 and 100 compared to the 40th lactation day. Furthermore, in the present study, litter size only affected the urea concentration, as it was significantly increased in the blood of ewes having twins (*p* = 0.043). At the same time, no significant interaction was found between lactation stage and litter size on the investigated metabolites in the blood of Dalmatian Pramenka ewes (*p* > 0.05). When compared with the reference values for sheep (Table 5), this study confirmed lower glucose concentrations and higher urea concentrations in the last two measurements (on the 70th and 100th days of lactation), and a slight increase in LDL-cholesterol in blood of Dalmatian Pramenka ewes.

The influence of lactation stage and litter size on the blood enzyme activities of lactating Dalmatian Pramenka ewes is shown in Table 8. Lactation stage had significant influence on ALT activity, as it was increased on the 70th day compared to the other two stages of lactation (*p* < 0.0001). Otherwise, this study proved no significant influence either of the litter size or the interaction between SL × LS on the activity of enzymes in the blood of Dalmatian Pramenka ewes during lactation (*p* > 0.05). When compared with the reference values for sheep (Table 5), in this study, higher GGT activity was found, and on the 70th day of lactation, there was a slightly higher ALT activity confirmed in the blood of the Dalmatian ewes.

## 4. Discussion

### 4.1. Influence of Lactation Stage and Litter Size on the Hemogram of Dalmatian Pramenka Ewes

Determination of the blood metabolic profile and hemogram provides valuable data on the animal’s energy status and on the metabolism of carbohydrates, fat, and protein [27,28]. Definition of the hemogram and blood metabolic profile of Dalmatian Pramenka ewes should be part of their breeding program because Dalmatian Pramenka sheep are usually kept in extensive systems, in which it is not possible to properly monitor the health status and nutrition of the sheep. Lactation is a demanding period in a sheep’s life, during which various changes in blood metabolic profile and hemogram may occur [6].

It should be noted that the blood reference values of sheep may vary depending on breed, production system, health status, nutritional management, physiological status and analytical methodology, and others. In the present study, deviations from the reference values were found for erythrogram and leukogram indicators [22], the average values of RBC, HGB, and MCHC were lower than the reference values, and the MCV and basophils were slightly higher. A normal distribution was also found only for the MCH content, while all other blood count indicators of the Dalmatian Pramenka ewes did not have a normal distribution, which was also adjusted for the statistical interpretation of the obtained results (Table 2; Figure 1). A comparison of the blood hemogram indicators in the present study was carried out with breeds with a similar production direction and breeding conditions. For example, Cincović et al. [29] did not determine the deviation of the mean value of hematological indicators from the reference values [22] in the blood of rams of the Bosnian Pramenka breed, but they determined a normal distribution for RBC and HCT. Shek-Vugrovečki et al. [30] determined a normal distribution for RBC, HGB, MCV, and MCH in the blood of Lika Pramenka sheep. Vojta et al. [31] concluded that by using smaller groups of sheep and applying appropriate statistical analyses, it is possible to correctly determine the reference interval of the studied blood parameters without the need to transform the original data obtained. Also, in their research on the development of blood reference intervals of Pelargonia Pramenka sheep, Dodovski et al. [32] determined a lower number of RBC and HGB, MCHC, and HCT contents at the lower limit compared to the reference values [22]. Also, the mentioned authors determined a normal distribution for MCV and HCT, while for other hemogram parameters, a non-normal distribution was determined. The mentioned changes in the hemogram parameters of Dalmatian Pramenka ewes indicate the need to develop reference values only for this breed, in order to better monitor the health of the herd in extensive production systems during the lactation period and to compare them with similar breeds of sheep.

In this study, many of the hemogram indicators of Dalmatian Pramenka ewes varied under the influence of lactation stage, which confirmed the research hypothesis. As lactation advanced, indicators of erythrogram (i.e., the content of HCT, RBC, as well as the content of HGB) in the ewes’ blood decreased significantly in the 3rd stage of lactation in comparison with the other two stages of lactation (Table 3). Leukogram indicators (i.e., WBC) were significantly higher in mid lactation (2nd stage of lactation) than in the 1st stage of lactation, while changes in leukocyte subpopulation, except monocytes, were not significant (Table 3). Reduction in total leukocytes in the blood of sheep at the early stage may be attributed to the increment in cortisol level (as a result of increased milk yield toward peak level), which is probably responsible for impairment of the cellular immune response [33]. The portion of monocytes was significantly higher in the 3rd stage of lactation compared to the 1st stage of lactation in Dalmatian Pramenka ewes. The 3rd stage of lactation (on the 100th day) showed significant deviation in most of the investigated erythrogram and leukogram indicators, which indicated that peak milk production caused various changes in sheep metabolism, as metabolic processes are engaged in milk production [34]. In the research performed in the Czech Republic, Konečná et al. [35] determined the highest DMY on the 94th day of lactation in Lacaune ewes, followed by the gradual decrease in DMY as lactation advanced. The same trend was described by Hernandez et al. [36]. Such findings are connected with the changes in the differential blood count, as there was an increase in WBC in our research on the 70th day of lactation, and a significant increase in monocytes on the 100th day of lactation. Similar changes in the increase in HGB and HCT content and the decrease in MCV, MCH, and lymphocytes with advancing lactation were found by Antunović et al. [6] in their research on Travnik Pramenka ewes. Similar changes were reported by El-Sherif and Assad [37], which were related to the decrease in RBC and the content of HGB in the blood of lactating ewes, especially during early lactation, which they linked to the occurrence of the hemodilution effect resulting from an increase in plasma volume and/or the increasing water mobilization to mammary glands through the vascular system. Namely, hemodilution represents an adaptive response to lactation resulting from the expansion of plasma volume and increased mammary blood flow, which facilitates the transport of water, nutrients, and the metabolic precursors required for milk synthesis. Consequently, the observed reductions in RBC, HGB, and HCT should be interpreted primarily as physiological adaptations supporting lactation. Moreover, the results published by Anwar et al. [38] also refer to the decreased RBC, HGB, and HCT contents. They argued that the reduced HCT in ewes’ blood in the first month of lactation pointed at the elevated RBC devastation in mammary cells, which was responsible for reduced HCT along with the mobilization of water to the mammary glands. Low values of HCT may indicate a high erythrocyte destruction by the active mammary glands of lactating ewes, low hemoglobin synthesis, and/or increasing water mobilization to the mammary glands through the vascular system [37,38] and may cause shortened erythrocyte lifespan and increased physiological erythrocyte turnover associated with intensive milk production. Compared with the reference values for sheep [22], the number of RBC, as well as the contents of HGB and MCHC were lower, while the contents of MCV and basophiles were higher in the blood of lactating Dalmatian Pramenka ewes (Table 2). Furthermore, it was determined that the content of HGB was below the reference range at all stages of lactation (Table 2), characterizing it as a relevant hematological deviation in Dalmatian Pramenka ewes, which justifies further investigation. Polizopoulous [39] stated that an increased MCV content indicates an enhanced regenerative response, while a decrease in MCHC indicates reticulocytosis or iron deficiency anemia. Moreover, this research confirmed the lower level of Fe in the blood of lactating Dalmatian Pramenka ewes (Table 5) when compared to the reference range determined by Kaneko et al. [24], which is 29.70–39.70 µmol/L. However, the mentioned reference values should be taken with certain caution because they are significantly influenced by many factors. For example, in research with Bosnian Pramenka rams, Cincović et al. [29] determined a lower hemoglobin content and number of erythrocytes in blood, which they associated with the fact that the erythrocytes of Pramenka breed rams are smaller than the erythrocytes of other breeds and species. Therefore, the mentioned authors point out that due to the clinical significance of anemia in sheep, it is also necessary to be aware of the morphometric properties of erythrocytes.

In this study, the influence of the litter size on the hemogram parameters of Dalmatian Pramenka ewes was not significant. Similar conclusions were reached by Kadhem and Al-Thuwain [12] in a study on Awassi sheep during lactation. They did not find significant changes in hematological parameters during a period of one month after lambing. This is also supported by the conclusions of Owens et al. [40], who emphasized that the content of hematocrit in sheep’s blood was not a precise indicator of the litter size influence.

### 4.2. Influence of Lactation Stage and Litter Size on the Blood Metabolic Profile of Dalmatian Pramenka Ewes

Blood reference values of most biochemical parameters, including the concentrations of investigated minerals, metabolites, and enzyme activity, may vary depending on breed, production system, health status, nutritional management, physiological status, and analytical methodology as well as others factors. In the present study, deviations from the reference values [24,25,26] were determined, namely lower average values were determined for the investigated concentrations of minerals (Ca, P-inorganic, Mg, and Fe) and glucose, and higher values of LDL-cholesterol and GGT activity, while the other parameters (12) were within the reference values. The highest variations were recorded in enzyme activity, which was to be expected. A normal distribution was also determined for 10 indicators of the blood metabolic profile of Dalmatian Pramenka ewes (Ca, P-inor., Mg, GUK, CHOL, LDL-CHOL, TP, ALB, GLOB, ALT; Table 5 and Figure 2), while the distribution was not normal for nine of the investigated indicators of the blood metabolic profile of Dalmatian Pramenka ewes, namely for Fe, urea, A/G ratio, TRIG, HDL-cholesterol, and enzymes (ALP, AST, GGT, and CK). The above was adapted to the statistical processing and interpretation of the obtained results. A comparison of the blood metabolic profile indicators of the subject research was carried out with breeds with similar production and rearing conditions. In a study conducted with Lika Pramenka sheep, Shek-Vugrovečki et al. [30] found a normal distribution for urea and TP, a non-normal distribution for GGT, ALB, CHOL, and a log-normal distribution for AST and GUK, while in a study with Bosnian Pramenka rams, Cincović et al. [29] determined a normal distribution only for some biochemical indicators in blood (ALB, GLOB and CHOL), but also higher mean values for GLOB and GLUC in blood compared to the reference values [24]. Furthermore, Dodovski et al. [32], when making blood reference values for Pelargonia Pramenka sheep, determined a lower mean concentration of Mg, a concentration of Ca at the lower limit, and a higher content of TRI, ALB, urea, and GGT in comparison with the reference values [24]. Also, the mentioned authors determined a non-normal distribution for GLUC, CHOL-HDL- and LDL-CHOL, TRIG, and P-inorganic. The mentioned changes in the blood metabolic profile indicators of Dalmatian Pramenka sheep obtained in the subject research indicate the need to conduct blood metabolic profile research in the future with the aim of creating our own reference values, especially in extensive breeding with highly demanding production phases such as lactation. The above will help in better monitoring of the health and nutritional status of this breed of sheep, as well as a comparison with other breeds with a similar production direction and environmental conditions of cultivation.

This study confirmed that many indicators of the blood metabolic profile of Dalmatian Pramenka ewes varied under the influence of the lactation stage, which confirmed the research hypothesis. This study confirmed that concentrations of Ca in the blood of Dalmatian Pramenka ewes significantly decreased as lactation advanced (*p* = 0.031), yet the concentration of P-inorganic increased (*p* < 0.0001; Table 6). In comparison with the reference values for Ca and P-inorganic, which, according to Kaneko et al. [24] range between 2.80–3.20 mmol/L and 1.62–2.60 mmol/L, respectively, Ca and P-inorganic values in this study were lower (Table 5). The Fe content in lactating Dalmatian Pramenka ewes’ blood decreased, and there were significant differences determined between the 1st and 3rd stages of lactation (*p* < 0.0001). The stated facts indicate the redirection of the metabolic pathways of these minerals, especially of Ca and P-inorganic, meaning that their transport from bones is increased due to increased milk synthesis during lactation. Although Ca and P have similar mobilization pathways from bones [41], Ca mobilization from blood into milk is stronger, even to a greater extent than P-inorganic [42], which can be linked to the results obtained in this study. Liesegang et al. [43] also confirmed reduced Ca levels in the blood of lactating ewes, which was connected with the increased Ca secretion through milk and its rearrangement in bones.

In this study, as lactation progressed, the concentration of glucose significantly decreased at 70 days and later returned to the initial values determined on the 40th day of lactation, whereas the concentrations of urea in Dalmatian Pramenka ewes’ blood increased during lactation (Table 7). Compared to the reference range determined by Kaneko et al. [24] for sheep, which for glucose is between 2.78 and 4.44 mmol/L, this study determined lower concentrations of glucose in lactating Dalmatian Pramenka ewes’ blood during all lactation stages (Table 5). Such findings indicate an energy imbalance identified in Dalmatian Pramenka ewes during lactation. Similar findings were published by Abdelsattar et al. [44] in their research on goats up to the 12th lactation week. Roubies et al. [45] also stated that the decrease in glucose concentration in the blood of lactating ewes was a consequence of its greater use for the synthesis of milk lactose with insufficient dietary supply to maintain blood glucose homeostasis. As Hyder et al. [46] pointed out, within extensive sheep farming systems, the adaptive mechanism of fermentative metabolism could lead to increased glucose utilization to maintain body functions, and could directly influence the reduction in serum glucose concentrations. The process of gluconeogenesis that takes place in the liver of ruminants is also a source of glucose in the blood, because it synthesizes glucose from non-carbohydrate substances such as amino acids, glycerol, lactic acid, and propionate. In ruminants, gluconeogenesis is particularly important because during the digestion process, most carbohydrates consumed in food are subjected to the action of the rumen microflora, which then ferments them to volatile fatty acids, namely acetic, propionic, and butyric acids, and only a small remainder of easily digestible carbohydrates from food escapes fermentation in the rumen and becomes a source of glucose for direct absorption into the blood [24]. Changes in the lipid profile in the blood of sheep during lactation may indicate a stronger effect of insulin and its lipolytic effect, but also a change in the quality of pasture during sheep grazing and the cereals/oats used in the diet (Table 1). Vernon et al. [47] found that the lipolytic effect of insulin was stronger in lactating sheep than in non-lactating sheep. Thus, the physiological adaptation associated with insulin is manifested as increased glucose production in the liver and reduced glucose utilization by tissues other than the mammary glands due to the increased need of the udder for glucose and milk synthesis. The modification of nutrient utilization after meal consumption at the beginning of lactation when the concentration of insulin in the blood is increased leads to a decrease in the redirection of nutrients to body fat/reserves and long tissues other than the mammary gland. This is especially pronounced if the supply of nutrients is insufficient and there is a greater mobilization of the body’s energy reserves to meet the needs due to increased milk synthesis, which is achieved by a stronger response to signals that stimulate lipolysis, leading to metabolic changes [48]. This is consistent with the results of our study. This is also supported by the conclusions of the study by Lunesu et al. [49], since in Sarda sheep during mid and late lactation, the concentration of growth hormone was low and insulin was high, which promotes the redistribution of nutrients toward peripheral tissues, especially when fed diets rich in starch. The authors also pointed out that contrary to the above, diets rich in digestible fiber stimulated the production of acetate, and were beneficial for milk yield and milk fat synthesis in Sarda sheep, with similar effects observed in East Friesian and Asaf sheep. In this study, the authors confirmed a significant increase in the blood urea concentrations of lactating Dalmatian Pramenka ewes. This can be connected with the lack of fermentable energy in the ration, due to which ammonia is used by microorganisms in the rumen for protein synthesis, which can then be taken by the rumen epithelium into the blood and liver, where they synthesize urea. Elevated blood urea levels in sheep are closely related to the production of ammonia in the rumen and its utilization by microbes that use volatile fatty acids (acetate, propionate, and butyrate) for energy, which mainly serve as the sheep’s primary energy source [50]. The study by Ljubičić et al. [51], which researched Dalmatian Pramenka lambs in similar farming conditions, also determined concentrations of urea (8.23 mmol/L) that were higher than the reference values. In the study on goats during lactation, Antunović et al. [52] also determined a significantly increased blood concentration of urea from the 15th to 90th day of lactation. In this study, the concentration of GLOB, was significantly increased, while the ALB concentration significantly decreased in the 2nd and 3rd stages of lactation (on the 70th and 100th lactation days; Table 7). Urea concentrations in sheep’s blood are a good indicator of protein supplies from feed [53]. It is known that ALB content shows the protein status of animals, whereas the TP content shows protein reserves in the animal body. A significant increase in urea concentrations on the 70th and 100th lactation days, with average concentrations being above the reference values (Table 7), may be related to progressive anemia in that period of lactation. This is in accordance with the research of Prabhakar and Mounika [54]. Increased GLOB content in blood indicates improved immune competence, which is crucial for protection against infections, while the decrease in blood ALB concentrations during lactation can be associated with a decrease in the crude protein content of pasture (Table 1). Antunović et al. [6] reported on similar changes in the increased concentrations of GLOB and TP in Travnik Pramenka ewes’ blood during lactation. The A/G ratio is used to determine changes in the blood protein levels, and it indicates the sheep’s immunity and nutrition during pregnancy and lactation [55]. In this study, the A/G ratio in ewes’ blood decreased as lactation progressed, which can be related to the increase in the content of globulin fractions that are required by the mammary gland for milk synthesis and antibody production [34]. Fitriani et al. [56] also reported that the A/G ratio was lower in lactating than in pregnant sheep.

As lactation advanced, most of the enzyme activities in the blood of Dalmatian Pramenka ewes during lactation were slightly increased, yet a significant increase was recorded only for ALT activities (*p* < 0.0001; Table 8). Determined changes in blood enzyme activities may indicate enhanced liver function in lactating ewes, with the aim of meeting the energy and protein needs for maintenance and milk production [44]. This is associated with more intense metabolic activities in the liver and the body reacting to a negative energy balance [57]. The above changes can be partially linked to changes in the chemical composition of the pasture (Table 1). In the research study by Djokovic et al. [58], an increase in the activity of the ALT enzyme in blood was also found in cows in the middle of lactation when it was the highest.

The effect of litter size on the blood metabolic profile of lactating Dalmatian Pramenka ewes was not significant in the present study, with the exception of significantly higher blood urea concentrations in ewes having twins compared with ewes with a single lamb. Similar findings were reported by Rhind et al. [14], who observed no significant effect of litter size on the overall mean blood concentrations of glucose, urea, total protein, and albumin in ewes during the first 10 weeks of lactation. These authors suggested that the metabolite levels associated with nutritional status might constitute a part of the driving force behind endocrine changes, which in turn influenced the availability of nutrients to the mammary tissue. Likewise, González-García et al. [59] did not prove a significant effect of litter size on blood glucose and triglyceride concentrations in multiparous ewes during lactation. The higher blood urea concentrations observed in Dalmatian Pramenka ewes with twins may indicate an energy imbalance in the diet, which is further supported by the reduced blood glucose concentrations recorded in the studied ewes.

Limitations of the study are the relatively small sample of sheep studied, the failure to perform a coprological examination of feces, and the inability to monitor pasture consumption on grazing sheep. In addition, future studies should include monitoring multiple sheep herds and determining some of metabolic hormones in blood (insulin, IGF-1, thyroxine), in order to develop our own blood reference values.

## 5. Conclusions

Based on the obtained results, it can be concluded that the lactation stage had a significant influence on the blood metabolic profile and hemogram, while an insignificant influence was recorded for the litter size of lactating Dalmatian Pramenka ewes. The flock, managed extensively on sub-Mediterranean pasture, showed, throughout lactation, a sustained energy deficit with a relative excess of degradable nitrogen, simultaneous deficiency of Ca, P, Mg, and Fe, and progressive anemia reaching day 100. This could be related to management specifics, particularly nutrition, or may indicate inherent breed characteristics of the ewes. The above results also indicate the need to include a larger number of herds in future research to develop our own blood reference values for this breed. When planning herd monitoring procedures, the determination of blood metabolic profile and hemogram should definitely be taken as routine herd-monitoring tools for indigenous sheep breeds raised under extensive production systems, especially during highly demanding production phases such as lactation, which is nutritionally very demanding for the animal.

## Figures and Tables

**Table 1:** Chemical composition of the feedstuffs.

Ingredient (g/kg DM)	Feedstuffs				
	Pasture			Hay	Oat
	40th	70th	100th		
Dry matter	941.5	955.7	963.7	948.3	942.7
Crude proteins	145.7	131.9	122.1	108.7	129.9
Ether extract	14.2	11.5	12.1	15.7	12.6
Crude fiber	149.1	155.0	162.8	302.9	100.8
Ash	109.8	101.0	100.1	65.0	54.3
NDF	301.8	335.5	381.3	535.9	495.2
ADF	208.2	253.2	280.2	380.8	215.8
Lignin	59.9	71.1	79.4	120.2	70.2

DM: dry matter; NDF: neutral detergent fiber; ADF: acid detergent fiber.

**Table 2:** Descriptive statistics, the 95% and 99% confidence intervals for lower and upper reference values and distribution for the hemogram indicators of lactating Dalmatian Pramenka ewes.

Indicators	Mean	Median	Sd	95% CI (Lower Limit–Upper Limit)	99% CI (Lower Limit–Upper Limit)	Normality Distribution	Reference Values *
RBC (×10^12^ L)	6.77	6.75	1.15	6.54–7.00	6.46–7.07	No	9–15
HGB, g/L	81.72	80.00	11.77	79.37–84.06	78.61–84.82	No	90–150
HCT, L/L	0.29	0.29	0.05	0.29–0.31	0.28–0.31	No	0.27–0.45
MCV, fL	47.99	43.20	13.93	39.22–56.75	36.39–59.59	No	28–40
MCH, pg	12.12	12.10	0.61	12.00–12.24	11.93–12.28	Yes	8–12
MCHC, g/L	279.89	277.00	22.37	275.42–284.32	273.97–255.76	No	310–340
WBC (×10^9^ L)	8.27	8.20	2.12	7.85–8.69	7.71–8.83	No	4–12 **
Eosinophyles, %	6.85	6.00	4.83	5.89–7.81	5.57–8.12	No	1.0–8.0
Neutrophiles, %	30.73	29.00	8.08	29.12–32.34	28.59–32.86	No	10.0–50.0
Lymphocytes, %	58.66	59.00	9.91	56.68–60.63	56.04–61.27	No	50.0–75.0
Basophils, %	1.97	2.00	1.37	1.70–2.24	1.61–2.33	No	0–1.0
Monocytes, %	1.80	2.00	1.95	1.51–2.09	1.42–2.18	No	0–4.0

CI—confidence interval; Sd—standard deviation; RBC (red blood cells); HGB (hemoglobin); HCT (hematocrit); MCV (mean corpuscular volume); MCH (mean corpuscular hemoglobin); MCHC (mean corpuscular hemoglobin concentration); WBC (white blood cells); * Baumgartner and Wittek [22]; ** Latimer et al. [23].

**Table 3:** Influence of lactation stage and litter size on the erythrogram of lactating Dalmatian Pramenka ewes.

Indicators	Stage of Lactation, Days (Mean)			SEM	*p* Values		
	40th	70th	100th		SL	LS	SL × LS
RBC (×10^12^ L)	7.29 ^a^	7.01 ^a^	6.00 ^b^	0.116	<0.0001	0.948	0.339
HGB, g/L	88.55 ^a^	83.36 ^a^	73.24 ^b^	1.183	<0.0001	0.660	0.297
HCT, L/L	0.32 ^a^	0.30 ^a^	0.27 ^b^	0.005	<0.0001	0.501	0.462
MCV, fL	56.49 ^a^	43.15 ^a^	44.32 ^a^	4.442	0.322	0.546	0.278
MCH, pg	12.30 ^a^	11.94 ^a^	12.13 ^a^	0.062	0.097	0.686	0.928
MCHC, g/L	285.52 ^a^	278.27 ^a^	275.82 ^a^	2.244	0.121	0.693	0.200

SL: lactation stage; LS: litter size; SL × LS: lactation stage × litter size; SEM—standard error of mean; RBC (red blood cells); HGB (hemoglobin); HCT (hematocrit); MCV (mean corpuscular volume); MCH (mean corpuscular hemoglobin); MCHC (mean corpuscular hemoglobin concentration); a, b: The same row means with different superscripts differ significantly at *p* < 0.05.

**Table 4:** Influence of lactation stage and litter size on the leukogram of lactating Dalmatian Pramenka ewes.

Indicators	Stage of Lactation, Days (Mean)			SEM	*p* Values		
	40th	70th	100th		SL	LS	SL × LS
WBC (×10^9^ L)	7.70 ^a^	9.14 ^b^	7.97 ^ab^	0.213	0.002	0.554	0.348
Differential blood smears, %							
Eosinophyles	7.79 ^a^	7.15 ^a^	5.61 ^a^	0.486	0.187	0.945	0.800
Neutrophiles	31.15 ^a^	30.91 ^a^	30.12 ^a^	0.812	0.807	0.552	0.880
Lymphocytes	57.70 ^a^	58.06 ^a^	60.21 ^a^	0.996	0.569	0.396	0.350
Basophils	2.12 ^a^	2.00 ^a^	1.79 ^a^	0.138	0.291	0.220	0.075
Monocytes	1.24 ^b^	1.88 ^ab^	2.27 ^a^	0.146	0.044	0.713	0.676

SL: lactation stage; LS: litter size; SL × LS: lactation stage × litter size; SEM—standard error of mean; WBC (white blood cells); a, b: The same row means with different superscripts differ significantly at *p* < 0.05.

**Table 5:** Descriptive statistics, the 95% and 99% confidence intervals for the lower and upper reference values and the distribution of the blood metabolic profile indicators of lactating Dalmatian Pramenka ewes.

Indicators	Mean	Median	Sd	95% CI (Lower Limit–Upper Limit)	99% CI (Lower Limit–Upper Limit)	Normality Distribution	Reference Values *
Ca, mmol/L	2.33	2.32	0.13	2.30–2.35	2.29–2.36	Yes	2.80–3.20
P-inorganic, mmol/L	1.29	1.33	0.14	1.26–1.39	1.24–1.41	Yes	1.62–2.60
Mg, mmol/L	0.89	0.88	0.09	0.87–0.90	0.86–0.91	Yes	0.91–1.31
Fe, µmol/L	17.05	17.34	4.61	16.43–18.27	16.13–18.56	No	29.70–39.70
Glucose, mmol/L	2.38	2.33	0.42	2.25–2.42	2.22–2.45	Yes	2.78–4.44
Cholesterol, mmol/L	1.78	1.80	0.30	1.74–1.88	1.72–1.88	Yes	1.35–1.97
HDL-cholesterol, mmol/L	1.16	1.18	0.20	1.14–1.22	1.13–1.23	No	1.00–1.18 ***
LDL-cholesterol, mmol/L	0.54	0.56	0.15	0.53–0.59	0.52–0.60	Yes	0.36–0.50 ***
Triglycerides, mmol/L	0.15	0.15	0.05	0.14–0.16	0.14–0.17	No	0.00–0.20
Urea, mmol/L	6.89	6.64	1.38	6.37–6.92	6.28–7.01	No	2.86–7.14
Total protein, g/L	64.07	63.99	4.68	63.06–64.93	62.76–65.23	Yes	60.00–79.00
Albumin, g/L	24.93	25.15	2.15	24.73–25.58	24.59–25.72	Yes	24.00–30.00
Globulin, g/L	39.45	38.84	4.83	37.87–39.80	37.56–40.11	Yes	35.00–57.00
Ratio A/G	0.63	0.66	0.12	0.64–0.68	0.63–0.69	No	0.30–1.00 **
AST, U/L	127.60	131.11	30.79	124.97–137.25	177.98–139.23	No	60–280
ALT, U/L	18.00	18.16	4.42	17.28–19.04	16.99–19.33	Yes	6–20
CK, U/L	98.20	105.33	39.96	37.36–113.30	94.78–115.82	No	35–280
GGT, U/L	59.20	57.36	12.07	54.98–59.72	54.18–60.55	No	20–52
ALP, U/L	134.50	149.08	92.56	130.62–167.84	124.64–173.52	No	68–387

CI—confidence interval; Sd—standard deviation; RBC (red blood cells); HGB (hemoglobin); HCT (hematocrit); MCV (mean corpuscular volume); MCH (mean corpuscular hemoglobin); MCHC (mean corpuscular hemoglobin concentration); WBC (white blood cells); * Kaneko et al. [24]; ** Očenaš et al. [25]; *** Antunović et al. [26].

**Table 6:** Influence of lactation stage and litter size on the blood mineral concentrations of lactating Dalmatian Pramenka ewes.

Indicators, mmol/L	Stage of Lactation, Days (Mean)			SEM	*p* Values		
	40th	70th	100th		SL	LS	SL × LS
Ca, mmol/L	2.38 ^a^	2.30 ^b^	2.29 ^b^	0.013	0.031	0.030	0.386
P-inorganic, mmol/L	1.04 ^c^	1.31 ^b^	1.61 ^a^	0.032	<0.0001	<0.0001	0.516
Mg, mmol/L	0.85 ^b^	0.88 ^ab^	0.92 ^a^	0.009	0.002	0.002	0.202
Fe, µmol/L	18.63 ^a^	19.11 ^a^	14.30 ^b^	0.464	<0.0001	0.662	0.516

SL: lactation stage; LS: litter size; SL × LS: lactation stage × litter size; SEM—standard error of mean; a, b, c: The same row means with different superscripts differ significantly at *p* < 0.05.

**Table 7:** Influence of lactation stage and litter size on the blood metabolite concentrations of lactating Dalmatian Pramenka ewes.

Indicators, mmol/L	Stage of Lactation, Days (Mean)			SEM	*p* Values		
	40th	70th	100th		SL	LS	SL × LS
Glucose	2.15 ^b^	2.68 ^a^	2.17 ^b^	0.042	<0.0001	0.745	0.964
Cholesterol	1.77 ^a^	1.89 ^a^	1.74 ^a^	0.030	0.134	0.474	0.512
HDL-cholesterol	1.13 ^a^	1.23 ^a^	1.17 ^a^	0.020	0.101	0.409	0.160
LDL-cholesterol	0.57 ^a^	0.58 ^a^	0.53 ^a^	0.015	0.467	0.796	0.768
Triglycerides	0.14 ^a^	0.18 ^b^	0.14 ^a^	0.005	0.0002	0.696	0.367
Urea	5.14 ^b^	7.31 ^a^	7.47 ^a^	0.139	<0.0001	0.043	0.102
Total protein, g/L	62.76 ^a^	64.42 ^a^	64.80 ^a^	0.471	0.222	0.635	0.848
Albumin, g/L	26.18 ^a^	24.96 ^b^	24.34 ^b^	0.216	0.008	0.352	0.423
Globulin, g/L	36.58 ^b^	39.46 ^a^	40.45 ^a^	0.485	0.008	0.374	0.887
Ratio A/G	0.73 ^a^	0.64 ^b^	0.61 ^b^	0.012	0.0006	0.202	0.803

SL: lactation stage; LS: litter size; SL × LS: lactation stage × litter size; SEM—standard error of mean; a, b: The same row means with different superscripts differ significantly at *p* < 0.05.

**Table 8:** Influence of lactation stage and litter size on the blood enzyme activities of lactating Dalmatian Pramenka ewes.

Enzymes, U/L	Stage of Lactation, Days (Mean)			SEM	*p* Values		
	40th	70th	100th		SL	LS	SL × LS
AST	129.87 ^a^	125.78 ^a^	137.67 ^a^	3.094	0.313	0.539	0.301
ALT	16.87 ^b^	20.79 ^a^	16.83 ^b^	0.444	<0.0001	0.492	0.076
CK	109.49 ^a^	92.18 ^a^	114.32 ^a^	4.016	0.269	0.268	0.115
GGT	53.94 ^a^	58.20 ^a^	59.95 ^a^	1.213	0.056	0.359	0.858
ALP	149.73 ^a^	152.83 ^a^	144.68 ^a^	9.302	0.885	0.436	0.479

SL: lactation stage; LS: litter size; SL × LS: lactation stage × litter size; SEM—standard error of mean; AST: aspartate aminotransferase; ALT: alanine aminotransferase; GGT: γ-glutamyl transferase; CK: creatine kinase; a, b: The same row means with different superscripts differ significantly at *p* < 0.05.

## Data Availability

The original contributions presented in this study are included in the article. Further inquiries can be directed to the corresponding author.

## Abbreviations

The following abbreviations are used in this manuscript:

DM	Dry matter
NDF	Neutral detergent fiber
ADF	Acid detergent fiber
SL	Stage of lactation
LS	Litter size
SL × LS	Stage of lactation × litter size
SEM	Standard error of mean
RBC	Red blood cells
HGB	Hemoglobin
HCT	Hematocrit
MCV	Mean corpuscular volume
MCH	Mean corpuscular hemoglobin
MCHC	Mean corpuscular hemoglobin concentration
WBC	White blood cells
CHOL	Cholesterol
HDL-cholesterol	High-density lipoprotein
LDL-cholesterol	Low-density lipoprotein
TRIG	Triglycerides
PHOS	P-inorganic
GUK	Glucose
GLOB	Globulin
ALB	Albumin
TP	Total protein
A/G	Albumin/globulin ratio
AST	Aspartate aminotransferase
ALT	Alanine aminotransferase
GGT	γ-Glutamyl transferase
CK	Creatine kinase
IGF-1	Insulin growth factor 1
